# Report of Mortality in Buffalo for the Month of Aug., 1856

**Published:** 1856-10

**Authors:** C. L. Dayton

**Affiliations:** Health Physician


ART. V.—*Report of Mortality in Buffalo for the month of Aug.*, 1856.
By C. L. Dayton, M. D., Health Physician.

| DISEASES. | No. | Males. | Females. |
|---|---|---|---|
| Accidens, | 5 | 4 | 1 |
| Asphyxia Immersorum, | 4 | 4 | 3 |
| Asthma, Hydrops, Diarrhœa et Hæmorrhagia Uterina, | 1 | | 1 |
| Ascites, | 1 | 1 | |
| Anasarca, | 1 | 1 | |
| Bronchitis, | 1 | 1 | |
| Cholera Morbus, | 3 | 2 | 1 |
| Cynanche Trachealis, | 1 | 1 | |
| Cholera Infantum, | 24 | 16 | 6 |
| Delirium Tremens, | 3 | 2 | 1 |
| Diarrhœa, | 23 | 6 | 16 |
| Dysentery, | 26 | 17 | 9 |
| Dentitio, | 4 | 2 | 2 |
| Debilitas Senilis, | 8 | 4 | 4 |
| Enteritis, | 3 | 1 | 2 |
| Erysipelas, | 1 | 1 | |
| Eclampsia, | 19 | 9 | 9 |
| Febris Typhus, | 2 | 2 | |
| " Typhoid, | 6 | 5 | 1 |
| Hydrocephalus, | 6 | 2 | 4 |
| Hydrops, | 2 | 2 | |
| Hyperæmia Cerebralis, | 1 | 1 | |
| " Pulmonalis, | 1 | 1 | |
| Ignotus, | 16 | 10 | 6 |
| Icterus, | 1 | 1 | |
| Marasmus, | 8 | 5 | 3 |
| Morbus Hepatis (organic), | 1 | 1 | |
| " Cordis, | 1 | 1 | |
| Meningitis, | 3 | | 3 |
| Mania Puerperarum, | 1 | | 1 |
| Pneumonitis, | 1 | | 1 |
| Paraplegia, | 1 | 1 | |
| Premature Birth, | 3 | 2 | |
| Pericarditis, | 1 | 1 | |
| Pertussis, | 2 | 2 | |
| Peritonitis, | 1 | | 1 |
| Summer Complaint, | 44 | 25 | 17 |
| Still-born, | 4 | 2 | |
| Suicide by hanging, | 1 | | 1 |
| Scrophula, | 2 | 2 | |
| Tuberculosis Pulmonalis, | 19 | 7 | 12 |
| Vomica, | 1 | 1 | |
| Yallow Ganders, or Rot-an Fevar,* | 1 | 1 | |
| Total, | 258 | | |

* Rot-an Fevar, or Yallow Ganders.

It strikes me this is a new disease, at least one heretofore unnoticed in this locality. As such it is, perhaps, of sufficient interest to the medical profession to warrant a special notice. I have given the name of the disease verbatim, as taken from the certificate of cause of death. The learned attendant seems to have been in doubt

### SEXES.

| | |
|---|---|
| Males, | 147 |
| Females, | 102 |
| Sex not given, | 9 |
| Total, | 258 |

### AGES.

| | | | |
|---|---|---|---|
| Still-born, | 4 | Between 10 years and 20 years, | 4 |
| 1 day, | 5 | " 20 " " 30 " | 17 |
| Between 1 day and 30 days, | 13 | " 30 " " 40 " | 18 |
| " 1 month and 6 months, | 43 | " 40 " " 50 " | 7 |
| " 6 months and 12 " | 64 | " 50 " " 60 " | 7 |
| " 1 year " 3 years, | 51 | " 60 " " 70 " | 6 |
| " 3 " " 5 " | 4 | " 70 " " 80 " | 2 |
| " 5 " " 10 " | 5 | " 80 " " 90 " | 2 |
| Total number under 10 years, | 189 | Total number over 10 years, | 63 |

Ages not given, 6 Total, 258

### NATIVITIES.

| | | | |
|---|---|---|---|
| American, (white, 118), colored, 4, | 122 | Prussian, | 0 |
| German, | 55 | French, | 2 |
| Irish, | 31 | Scotch, | 1 |
| English, | 9 | Italian, | 2 |
| Canadian, | 3 | Country not given, | 33 |

Total, 258

which of the diseases named asphyxiated his patient. Perhaps while mystified in the vain attempt to form a correct diagnosis, he originated an erroneous nomenclature. If it should read, Rotten Ganders, or Yellow Fever, then by reasoning by exclusion, we might arrive at the true point; for the latter disease most certainly has not made its appearance in Buffalo, therefore if it was either, we should say it must have been the former; and we can readily conceive that the consumption of "rotten ganders," or any other putrid meat during dog days, would produce death; consequently this patient probably succumbed from the effects of rotten ganders, unless the profound Dr. was mistaken in regard to the sex of the fowl.

If horse doctors, who are permitted to practice upon the human species, would, when they kill a patient uniformly send in a blank certificate, our record of diseases would be much more perfect.

